# Prof. L. W. Mauthner

**Published:** 1858-07

**Authors:** 


PROFESSOR MAUTHNER, RITTER VON MAUTHSTEIN, Director of the St. Anne's Children's Hospital, Vienna, recently died of meningitis, in the prime of life. He was an accomplished and hospitable gentleman, and always took great pleasure in showing kind attentions to Americans stopping at, or passing through, the Austrian Capital. He was well known as an author, having written on "The Medicinal Power of the Cold Water Douche and Mineral Waters," and on the "Diseases of the Brain and Spinal Cord of Children." His last production was the "Diatetics of Children," which has gone through several editions, and has met with a French translation. Periodical Medical Literature owes much to his pen, having contributed largely to his *Journal für Kinderkrankheiten.*

